# A homozygous nonsense mutation in *DCBLD2* is a candidate cause of developmental delay, dysmorphic features and restrictive cardiomyopathy

**DOI:** 10.1038/s41598-021-92026-0

**Published:** 2021-06-18

**Authors:** Kheloud M. Alhamoudi, Tlili Barhoumi, Hamad Al-Eidi, Abdulaziz Asiri, Marwan Nashabat, Manal Alaamery, Masheal Alharbi, Yazeid Alhaidan, Brahim Tabarki, Muhammad Umair, Majid Alfadhel

**Affiliations:** 1 Medical Genomics Research Department, King Abdullah International Research Center (KAIMRC), King Saud Bin Abdulaziz University for Health Sciences, King Abdulaziz Medical City, Ministry of National Guard Health Affairs, Riyadh, Kingdom of Saudi Arabia; 2 Medical Core Facility and Research Platforms, King Abdullah International Research Center (KAIMRC), King Saud Bin Abdulaziz University for Health Sciences, King Abdulaziz Medical City, Ministry of National Guard Health Affairs, Riyadh, Kingdom of Saudi Arabia; 3 Faculty of Applied Medical Sciences, University of Bisha, Al Nakhil, 225, Bisha, 67714 Kingdom of Saudi Arabia; 4 Division of Genetics, Department of Pediatrics, King Abdullah Specialized Children’s Hospital, King Abdulaziz Medical City, Ministry of National Guard Health Affairs, P.O Box 22490, Riyadh, 11426 Kingdom of Saudi Arabia; 5 Developmental Medicine Department, King Abdullah International Medical Research Center, King Saud Bin Abdulaziz University for Health Sciences, King Abdulaziz Medical City, Ministry of National Guard Health Affairs, Riyadh, Kingdom of Saudi Arabia; 6 Division of Pediatric Neurology, Department of Pediatrics, Prince Sultan Military Medical City, Riyadh, Kingdom of Saudi Arabia

**Keywords:** Genetics, Molecular biology

## Abstract

*DCBLD*2 encodes discodin, CUB and LCCL domain-containing protein 2, a type-I transmembrane receptor that is involved in intracellular receptor signalling pathways and the regulation of cell growth. In this report, we describe a 5-year-old female who presented severe clinical features, including restrictive cardiomyopathy, developmental delay, spasticity and dysmorphic features. Trio-whole-exome sequencing and segregation analysis were performed to identify the genetic cause of the disease within the family. A novel homozygous nonsense variant in the *DCBLD2* gene (c.80G > A, p.W27*) was identified as the most likely cause of the patient’s phenotype. This nonsense variant falls in the extracellular N-terminus of *DCBLD2* and thus might affect proper protein function of the transmembrane receptor. A number of in vitro investigations were performed on the proband’s skin fibroblasts compared to normal fibroblasts, which allowed a comprehensive assessment resulting in the functional characterization of the identified *DCBLD2* nonsense variant in different cellular processes. Our data propose a significant association between the identified variant and the observed reduction in cell proliferation, cell cycle progression, intracellular ROS, and Ca2 + levels, which would likely explain the phenotypic presentation of the patient as associated with lethal restrictive cardiomyopathy.

## Introduction

Cardiomyopathy is a cardiovascular disease (CVD) characterized by weakening of the heart muscle. It encompasses a range of types, such as hypertrophic cardiomyopathy (HCM), dilated cardiomyopathy (DCM) and restrictive cardiomyopathy (RCM)^1^. Although cardiomyopathies are considered a relatively common CVD, RCM is one of the least common types^2^. This rare type is characterized by enlargement of the ventricles, which impairs the heart muscle from contracting and relaxing effectively (diastolic dysfunction)^3^. Impaired muscle relaxation causes blood to back up in the atria and lungs, which reduces the amount of blood in the ventricles. Thus, they completely fill with blood between heartbeats^3^. All types of cardiomyopathies can lead to atrial tachycardia and blood clots and may cause heart failure^1^.

The molecular aetiology of nearly all of these CVDs is a genetic predisposition, and most are heritable^4,5^. Considerable progress in recent genomics applications, especially the development of high-throughput next-generation sequencing, has been tremendously advantageous and enabled the identification of the prevalence of familial heritable variants that play a role in conferring cardiomyopathies, which has been continually growing, especially for DCM and HCM^6–11^. However, the identification of the genetic causes of RCM is still limited when compared to other cardiomyopathies due to its rare incidence rate^12,13^. Mutations in genes such as the troponin T (*TNNI2*)^14^, troponin I (*TNNI3*)^15^, alpha-cardiac actin (*ACTC*)^16^ and beta-myosin heavy chain (*MYH7*)^17^ genes have been shown to be a major cause of familial RCM. However, some people with familial RCM do not have an identified mutation in any of the known associated genes. Therefore, a large unmet need remains for the identification of variants that are associated with RCM.

*DCBLD2 encodes* discoidin, CUB, and LCCL domain-containing protein 2. It is a gene residue on chromosome 3q12.1 (NM_080927.4) chr3:98,795,941–98,901,695. It is also abbreviated as endothelial and smooth muscle cell-derived neuropilin-like protein (ESDN) and/or Charcot–Leyden crystal protein pseudogene 1 (CLCP1). The *DCBLD2* gene is a type-I transmembrane receptor that structurally contains the complement Clr/Cls, Uegf, Bmp1 (CUB) domain (114 amino acids (a.a)), limulus clotting factor C, Coch-5b2, and Lgl1 (LCCL) domain (98 a.a), the factor V/VIII coagulation homology domain in the extracellular N-terminus, and a repeat domain in the intracellular C-terminus^18^. Therefore, it bears structural similarities to neuropilin-like transmembrane scaffolding receptors, such as vascular endothelial growth factor (VEGF) receptors and semaphorins, which have a wide variety of functions in developmental processes and vascular and tumour biology^19^.

It has been demonstrated that the *DCBLD2* gene is highly conserved among mammals and is expressed in the brain, nerve bundles, testis, heart, skeletal muscle, and cultured vascular smooth muscle cells^19^. O’Conner et al. identified *DCBLD2* as a novel platelet membrane receptor^20^. DCBLD2 has been found to participate in critical roles in vascular remodelling^18^, influencing vascular smooth muscle cell (VSMC) proliferation^18,19^, cell motility and metastasis of some cancers^21–23^ and neuronal positioning^24^. Although its role in the regulation of cell growth is known, little is known about the signalling features of *DCBLD2* that drive its molecular activity. To date, no pathogenic variants in *DCBLD2* have been reported in association with a specific human disease.

In this study, we report the identification and functional characterization of a novel homozygous nonsense mutation in exon 1/16 of the *DCBLD2* gene (c.80G > A, p.W27*) in a Saudi patient presenting with a multisystem disorder including RCM, developmental delay, spasticity and dysmorphic features.

## Materials and methods

### Human subject

The proband underwent a full routine clinical evaluation, including familial history, echocardiogram and radiological examinations, at King Abdulaziz Medical City in Riyadh, Saudi Arabia. In addition, several genetic evaluations, such as whole-exome sequencing (WES) and mitochondrial genome testing, were conducted at GeneDx-certified clinical diagnostic laboratory with expertise in rare and ultrarare genetic disorders (Galthersburg, USA, https://www.genedx.com).

### Ethical statement

Ethical approval of this study was obtained from King Abdullah International Medical Center (KAIMRC) according to the Institutional Review Board (IRB), study number (RC18/017/R). The standard informed clinical consent form was obtained and signed by the index’s parents. The study was conducted in accordance with the tenets of the Declaration of Helsinki. Additionally, written informed consent was obtained from the index patient’s parents for publication.

### Sample collection and DNA extraction

To assess the multisystem disorder, blood samples were collected from the affected proband and her parents in EDTA tubes for DNA extraction. Genomic DNA was isolated using a QIAamp Blood Midi Kit (Qiagen) according to the manufacturer’s instructions.

### Whole-exome sequencing

A trio of WES analyses of the proband and both her parents was performed at a GeneDx-certified clinical diagnostic laboratory (Galthersburg, USA, https://www.genedx.com). The sequencing methodology and variant interpretation protocol have been previously described^25^. Briefly, the Agilent Clinical Research Exome kit was used to target the exonic regions and flanking splice junctions of the genome. These targeted regions were sequenced simultaneously on an Illumina HiSeq 2000 sequencing system with 100-bp paired-end reads. Bidirectional sequences were assembled and aligned to reference gene sequences based on the human genome build GRCh37/UCSC hg19 (http://genome.uscs.edu/).

### Whole-exome sequencing filtration steps

Data analysis and interpretation for WES data for the proband (IV-3) and her parents (III-3 and III-4) were also performed by GeneDx’s XomeAnalyzer using a custom-developed bioinformatics pipeline. Briefly, primary filtering was performed using standard methods, including filtering out low-quality reads and potential artefacts. Subsequently, variant classification was performed using guidelines similar to the published American College of Medical Genetics and Genomics (ACMGG) guidelines^26^. All phenotype-driven gene lists were reported in the Human Phenotype Ontology, and the Human Gene Mutation Database, ClinVar, 1000 Genomes database, NHLBI GO Exome Sequencing Project, OMIM and PubMed databases were considered to evaluate genes and detect sequence changes of interest. All pertinent inheritance patterns were considered. The family history and patient clinical information provided were used to evaluate the variants that were eventually identified in genes associated with intracranial haemorrhage, subcortical cysts, brain iron accumulation, corneal clouding, anaemia, thrombocytopenia, hydronephrosis, restrictive cardiomyopathy, developmental delay, spasticity and dysmorphic features. All identified variants were evaluated with respect to their pathogenicity and causality. The general assertion criteria for variant classification are publicly available on the GeneDx ClinVar submission page (http://www.ncbi.nlm.nih. gov/clinvar/submitters/26,957/). All variants related to the phenotype of the patient, except for benign or likely benign variants, were reported. Eventually, Sanger sequencing was used to confirm all identified variants in this proband and all available members of the family for variant segregation analysis. Sequence alterations were reported according to the Human Genome Variation Society (HGVS) nomenclature guidelines^27^. The Encyclopedia of DNA Elements (ENCODE) annotation on the UCSC genome browser (http://genome.ucsc.edu/) was used to confirm and annotate the variants predicted in this analysis. Furthermore, transcription factor binding site annotation for each locus was determined using the Jasper database accessible at http://jaspar.genereg.net^28^.

### Mitochondrial genome testing

Deletion testing of the mitochondrial genome was analysed at GeneDx. Briefly, the entire mitochondrial genome from the submitted samples was amplified and sequenced using solid-state sequencing by a synthesis process. The DNA sequence was assembled and analysed in comparison with the revised Cambridge Reference Sequence (rCRS)^29^ and the reported mutations and polymorphisms listed in the human mitochondrial genome (MITOMAP) database (http://www.mitomap.org)^30^. The presence of a disease-associated sequence variant, if present, would be confirmed by Sanger sequencing. A reference library of more than 6000 samples from different ethnic groups and online databases for mtDNA variations was used to evaluate variants of unknown clinical significance.

### Tissue samples and cell lines

Skin fibroblasts from the proband were obtained with informed consent, and their use was approved by the internal review board at King Abdulaziz Medical City in Riyadh, Saudi Arabia. A normal fibroblast cell line (HS27) that contained wild-type DCBLD2 was purchased from ATCC (USA) and used as a normal control. Both cell lines were cultured in Chang D medium (Irvine Scientific) supplemented with 10% foetal bovine serum (Gibco) and 1% penicillin/strep antibiotic (Gibco) and incubated in a humidified atmosphere of 5% CO_2_ and 95% air.

### Proliferation assay

Cells at the exponential growth phase were harvested and seeded at 10,000 cells per well in flat-bottom microtiter plates (Nunc, Fisher Scientific). Cell proliferation was assessed by a colorimetric assay using 3-(4,5-dimethylthiazol-2-yl)-2,5-diphenyltetrazolium bromide (MTT) dye (Promega) according to the manufacturer’s instructions. Briefly, 10% MTT solution was added to each well and incubated for 4 h. Then, 100 µl of SDS was added, and the cells were incubated for another 30 min. The absorbance value was read spectrophotometrically at 540 nm (SpectraMax M5, Molecular Devices). This was performed in at least six technical replicates and at least triplicate biological repeats. Mean absorbance readings were calculated for each biological repeat and expressed as a percentage of controls.

### Cell cycle analysis

The cell cycle was assessed by propidium iodide (PI) staining (Life Technologies) and fluorescence-activated cell sorting (BD Facscanto II flow cytometer, BD Biosciences). Cells were harvested and collected by centrifugation at 200* g* for 3 min. The cells were fixed using ice-cold 70% ethanol in 1X PBS (Gibco) and then stored at -20 °C overnight. After incubation, the cells were washed, centrifuged, treated with 10 μg/ml RNase A (Invitrogen) and resuspended in PBS containing 50 μg/ml PI buffer solution. Control cells were prepared without staining, and data were acquired for unstained cells. At least 20,000 cells were analysed in one parameter mode, and the calculations were carried out using FACSDiva software Version 6.1.3 (BD Biosciences) for cell cycle analysis.

### Reactive oxygen species measurement

Flow cytometric analysis for ROS detection was performed as previously described^31,32^ with slight modifications. Briefly, cells were stained using 10 µl of the permeable fluorogenic probe 2’,7’-dichlorodihydrofluorescein diacetate (DCF-DA) (Thermo Fisher Scientific) in the dark at 37 °C for 30 min. Cells were prepared without staining, and data were acquired for unstained cells, control and proband samples. Reactive oxygen species (ROS) activity and green fluorescence intensity within a cell were measured and analysed with a flow cytometer (excitation 488 nm/emission 530 nm). The fluorescence intensity was measured because the results are proportional to the ROS levels within the cell.

### Intracellular calcium assessment

Intracellular calcium (Ca2 +) was determined by Fluo-4/AM fluorescence (Biotium, Fremont, USA). The membrane-permeant Fluo4/AM ester is hydrolysed intracellularly by esterases into Fluo-4. Fluorescence increases after binding to Ca2 + ions, which serve as an indicator of the Ca2 + cellular content. Cells were washed in Ringer buffer (5 mM CaCl_2_) and incubated with 5 mM Fluo4/AM at 37 °C. After 30 min, the cells were washed twice to remove excess stain, resuspended in 300 ml of 5 mM CaCl_2_ Ringer solution and analysed by a BD FACSCanto II at excitation 488 nm/emission 530 nm.

### Microscopy

Immunofluorescence microscopy was performed as previously described with modifications^33^. The EVOS FL Auto system (Thermo Fisher Scientific) was used to visualize intracellular calcium localization by Fluo-4-AM staining in proband fibroblasts and HS27 normal cells.

### Statistical analysis

Statistical analysis was performed using GraphPad Prism version 8. The data are presented as the mean ± standard deviation (SD) of at least three biological replicates or as indicated. The data were analysed using Student’s unpaired *t-*test between the primary proband’s fibroblasts and normal fibroblasts. Statistical significance was represented by a P-value ≤ 0.05.

## Results

### Phenotypic presentation

**Figure 1:**
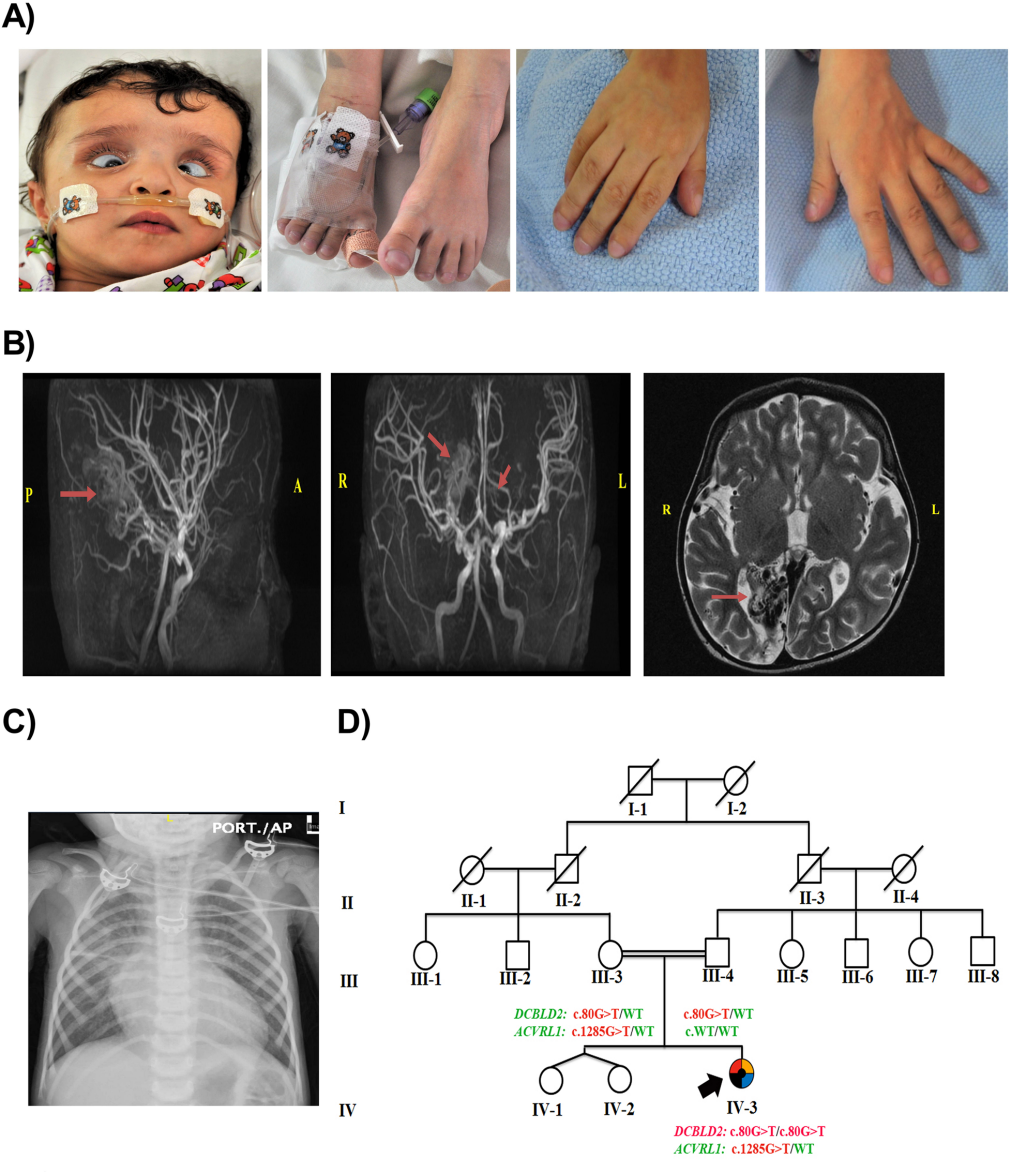
The index’s case presentation and genetics pedigree information. (**A**) Clinical features of the proband showing dysmorphic features, including frontal bossing, hypertelorism, deep-seated eyes, bilateral strabismus, corneal opacity, depressed nasal bridge, upturned nose, micrognathia, low-set ears, brachycephaly, and high arched palate. The hands and feet appeared small with broad thumbs. (**B**) Brain MRI and MRA: MRA (sagittal and coronal sections) showing right posterior cerebral and small left posterior-central gyrus arteriovenous malformations. (**C**) Chest X-ray showing cardiomegaly and congestion of the pulmonary vessels. T2-weighted axial section MRI shows right occipital cystic encephalomalacia. (**D**) Pedigree of the consanguineous Saudi family who had a daughter affected with a (black square) Restrictive cardiomyopathy, (blue square) Thrombocytopenia, (red square) Spasticity, (yellow square) Developmental delay and (black circle) Dysmorphic features; pedigree constructed from the details provided by the index’s mother, generated using (https://www.progenygenetics.com); squares (males); circles (females); slashed symbols (deceased individuals); annotated symbols (affected individuals); open symbols (unaffected individuals); arrowheads (index).

The proband (IV-3) was a 5-year-old Saudi female patient whose mother experienced an uneventful pregnancy and a spontaneous vaginal delivery at 36 weeks and 5 days. There was a prolonged rupture of the membranes with meconium-stained liquor. The proband was delivered flat, and suction was performed immediately. The birth weight was 2.5 kg, and the APGAR score was 7 and 9 at 1 and 5 min, respectively. Upon delivery, she was noted to have dysmorphic features in the form of wide skull sutures, low-set ears, low hairline, deep-seated eyes, and bilateral cloudy corneas (Fig. 1A).

At the age of 2 months, the patient (IV-3) presented to the emergency room for respiratory distress and cyanosis. She was admitted to the hospital and was diagnosed with bronchiolitis. The patient also demonstrated fluctuating haemoglobin levels and platelet counts during admission. Bone marrow aspiration showed depleted iron stores, with an adequate number of megakaryocytes. A second ophthalmology assessment was performed and revealed corneal dystrophy, and the patient was given cyclopentolate eye drops. Later, the patient suffered recurrent chest infections and developed recurrent attacks of tachycardia, for which Holter monitoring was performed. This revealed frequent attacks of narrow and wide complex tachycardia with T wave alteration.

At the age of 13 months, the patient showed respiratory distress, cyanosis, and engorgement of the neck veins. She developed bronchial asthma exacerbation associated with tachycardia and atrial fibrillation. Her heart rate reached up to 300 beats per minute. She had elevated blood pressure, generalized oedema, liver engorgement, and slight renal function impairment. Her growth parameters were as follows: length of 78 cm (75th percentile); weight of 9 kg (10th–25th percentile); and head circumference of 46 cm (25th percentile). She had dysmorphic features, including frontal bossing, hypertelorism, deep-seated eyes, bilateral strabismus, corneal opacity, depressed nasal bridge, upturned nose, micrognathia, low-set ears, brachycephaly, and high arched palate. The hands and feet appeared small with broad thumbs, Mongolian spots were present on the back, and she had bilateral lower limb spasticity (Fig. 1A).

At the age of 5 years, the patient continued to suffer from developmental delay and failure to thrive. All her growth parameters fell below the third percentile. She developed tonic seizures after a febrile illness. Additionally, her thrombocytopenia had worsened and required recurrent platelet transfusion. The patient developed gastrointestinal bleeding and could not tolerate enteral feeding. The patient ultimately experienced multiple organ failure and intractable respiratory and metabolic acidosis and unfortunately died.

### Clinical and genetic evaluation

Due to the multisystem disorder and phenotypic complexity, the patient underwent an extensive radiological, haematological, metabolic, and genetic workup. The postnatal radiological investigations included neonatal head ultrasound and brain magnetic resonance imaging (MRI). Both revealed bilateral subcortical frontal small intracerebral haematoma with no mass effect. The abdominal ultrasound was negative except for grade 1 hydronephrosis. Her latest brain MRI showed large right posterior cerebral and small left posterior-central gyrus arteriovenous malformations (AVMs). In addition, she had left superior parietal and right occipital cystic encephalomalacia and a small old infarct in the right posterior thalamus (Fig. 1B). The initial cardiac echo showed small patent ductus arteriosus (PDA). Cardiac electrocardiography (ECG) showed premature supraventricular complexes, left ventricular hypertrophy, and borderline QT interval. An echocardiogram showed dilation of both atria with atrioventricular valve regurgitation, which was suggestive of RCM (Fig. 1C). An abdominal ultrasound showed a cirrhotic liver with multiple hypoechoic focal lesions.

The ophthalmological assessment showed bilateral retinal haemorrhages. The initial investigations showed a low platelet count, reaching 68, but it improved spontaneously, peaking at 123. The peripheral blood smear showed microangiopathic haemolytic anaemia and basophilic stippling with increased reticulocyte count. Bone marrow aspiration was performed, which showed increased megakaryocytes, which supported the peripheral cause of her thrombocytopenia. All other haematological and biochemical investigations were unremarkable.

Similarly, all the metabolic investigations requested for the patient were unremarkable, including tandem mass spectrometry, new-born screening, urine organic acids, plasma amino acids, urine for mucopolysaccharides, oligosaccharides, very-long-chain fatty acids, purines/pyrimidines, lactic acid, and ammonia levels.

The proband is from parents who are first cousins, consanguineous, and asymptomatic, and they have two healthy daughters (twins) (IV-1 and IV-2) (Fig. 1D). The pedigree revealed a recessive inheritance pattern, the patient had a normal karyotype, and no mutations associated with mitochondrial metabolism disorders were identified by the analysis of the entire mitochondrial genome.

### Identification of a novel DCBLD2 variant

To identify the causative variant contributing to the proband’s phenotype, WES was performed for the proband (IV-3) and her parents, as described previously^25^. Filtration of the identified variants was performed by the DNA diagnostic expert laboratory at GeneDx considering all patterns of inheritance^26^. Only pathogenic, likely pathogenic, and non-synonymous (NS) variants causing missense, nonsense, frameshift, splice site variants (SS), coding insertions, or deletions (indel) were identified. The outcomes of WES data analysis and the filtration steps resulted in the identification of two variants, namely, a heterozygous variant in *ACVRL1* (c.1285 G > T, p.V429L (GTG > TTG)) and a homozygous nonsense variant in *DCBLD2* (c.80 G > A, p.W27* (TGG > TAG)), associated with the patient’s fatal phenotype.

The pathogenic variant (c.1285 G > T) is found in exon 9/10 of the *ACVRL1* gene (NM_000020.2), Chr12 (GRCH37): g.52312807. ACVRL1 encodes a serine/threonine-protein kinase receptor R3 that is part of a family of cell-surface receptors for the TGF superfamily of ligands (MIM 601,284). The V429L variant in the *ACVRL1* gene has not been published as a mutation, nor has it been reported as a benign polymorphism. The V429L variant was found to have an autosomal dominant pattern inherited from the proband’s mother (III-3). The mother was healthy except for long-standing anaemia and easy fatigability, which were reported to be associated with hereditary haemorrhagic telangiectasia type 2 (HHT2)^34^. This could explain the AVM discovered in the patient’s brain MRI as well as liver cirrhosis and hypoechoic focal lesions in the liver. The V429L variant occurs at a position where the amino acid valine is highly conserved across species. In silico analysis predicts that this variant is probably damaging to the protein structure and function. The V429L variant is a strong candidate for a disease-causing mutation that may be associated with the patient’s reported intracranial haemorrhage, anaemia, and thrombocytopenia.

However, the novel homozygous nonsense variant (c.80 G > A) in exon 1/16 of the *DCBLD2* gene (NM_080927.3) Chr3 (GRCH37): g.98514785, which causes an amino acid change from tryptophan to a stop codon (TGG > TAG) at position 27 (p.W27*), was also identified. To our knowledge, the W27* variant in the *DCBLD2* gene has not been reported previously as a disease-causing mutation or as a benign polymorphism. As verified by Sanger sequencing performed by GeneDx, the parents and siblings were evaluated using segregation analysis, which showed that all were heterozygous for the identified *DCBLD2* variant. Therefore, the W27* variant in the *DCBLD2* gene was considered the most likely candidate for a disease-causing variant with a potential relationship with the patient’s phenotype, including RCM, developmental delay, spasticity, and dysmorphic features. This finding was further evaluated in multiple cellular processes.

### In silico classification of the identified DCBLD2 variant

**Figure 2:**
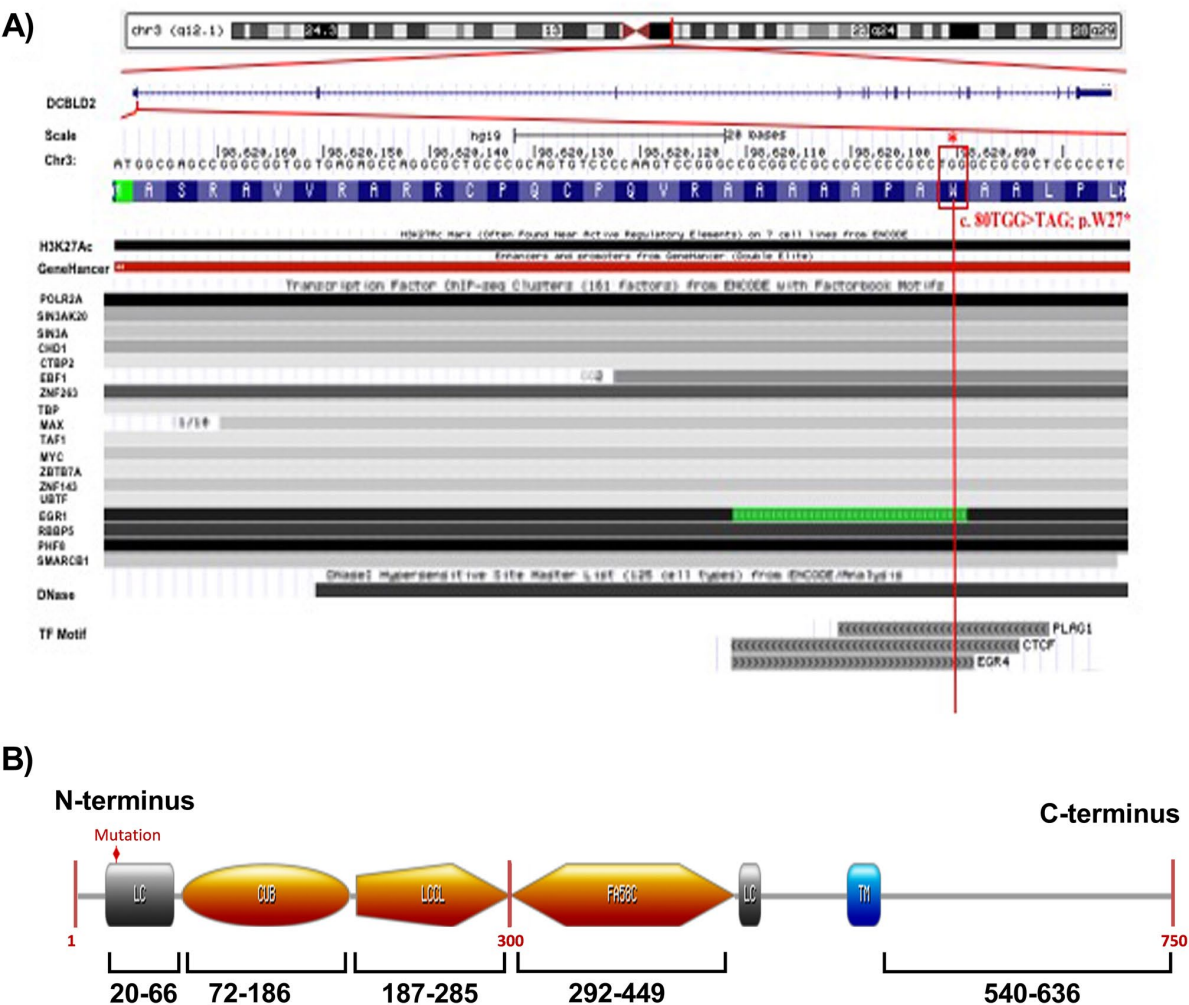
Schematic diagram and annotation of the identified c.80G > A, p.W27* variant in the *DCBLD2* gene. (**A**) The identified nonsense variant in the *DCBLD2* gene (c.80G > A; p.W27*) is located on chromosome 3 q12. The variant falls in the first exon and in some of the ENCODE functional data tracks on the hg19 in the UCSC genome browser. The variant falls in a region with strong regulatory elements including sites of DNaseI hypersensitivity, chromatin Imunoprecipitation (CHIP-seq) and predicted multiple transcription factor binding sites (TFBSs) such as PLAG1, CTCF and EGR4; the darkness of the segment is proportional to the signal strength. The arrows the strand direction. (**B**) The identified variant that creates a stop codon at amino acid 27 is located on the N-terminus of DCBLD2. The diagram was generated using prosite (https://prosite.expasy.org/cgi-bin/prosite).

An in silico analysis and alignment of this identified variant was performed using some ENCODE functional annotation on the UCSC genome browser. This annotation was focused on aligning the identified variant with multiple ENCODE data tracks including transcriptional and regulatory information such as DNaseI hypersensitivity (DNase-seq), Chromatin Imunoprecipitation (ChIP-seq) and DNase footprinting. As illustrated in Fig. 2A, It was revealed that the identified variant 80G > A; p.W27* in the *DCBLD2* gene overlaps a region with strong regulatory evidence including promoters, enhancers and DNaseI hypersensitivity that is largely restricted and specific to 125 cell types. The DNaseI hypersensitivity peak suggests an unwounded chromatin region in H3K27Ac. The ChIP-seq peaks suggest the presence of multiple transcription factor binding sites (TFBSs) in this region from multiple independent cell lines. Further computational predictions using regulatory information from the Jaspar database (http://jaspar.genereg.net) has performed, such as the presence of position weight matrices (PWM) and TF binding motifs^28^. It was revealed that the identified variant falls and might disrupt potential transcription factors binding sites such as PLAG1, CTCF and EGR4. Additionally, the variant is predicted to fall in an active regulatory site of the N-terminal region of the *DCBLD2* gene (Fig. 2B). This possibly has a major consequence on the *DCBLD2* transmembrane receptor’s function. The STRING protein interaction shows that there is a strong interaction between DCBLD2 and several other neuropilin proteins, such as semaphorins, VEGF and BAMBI.

### Determination of the effect of the identified W27* on cell cycle progression in patient fibroblasts

**Figure 3:**
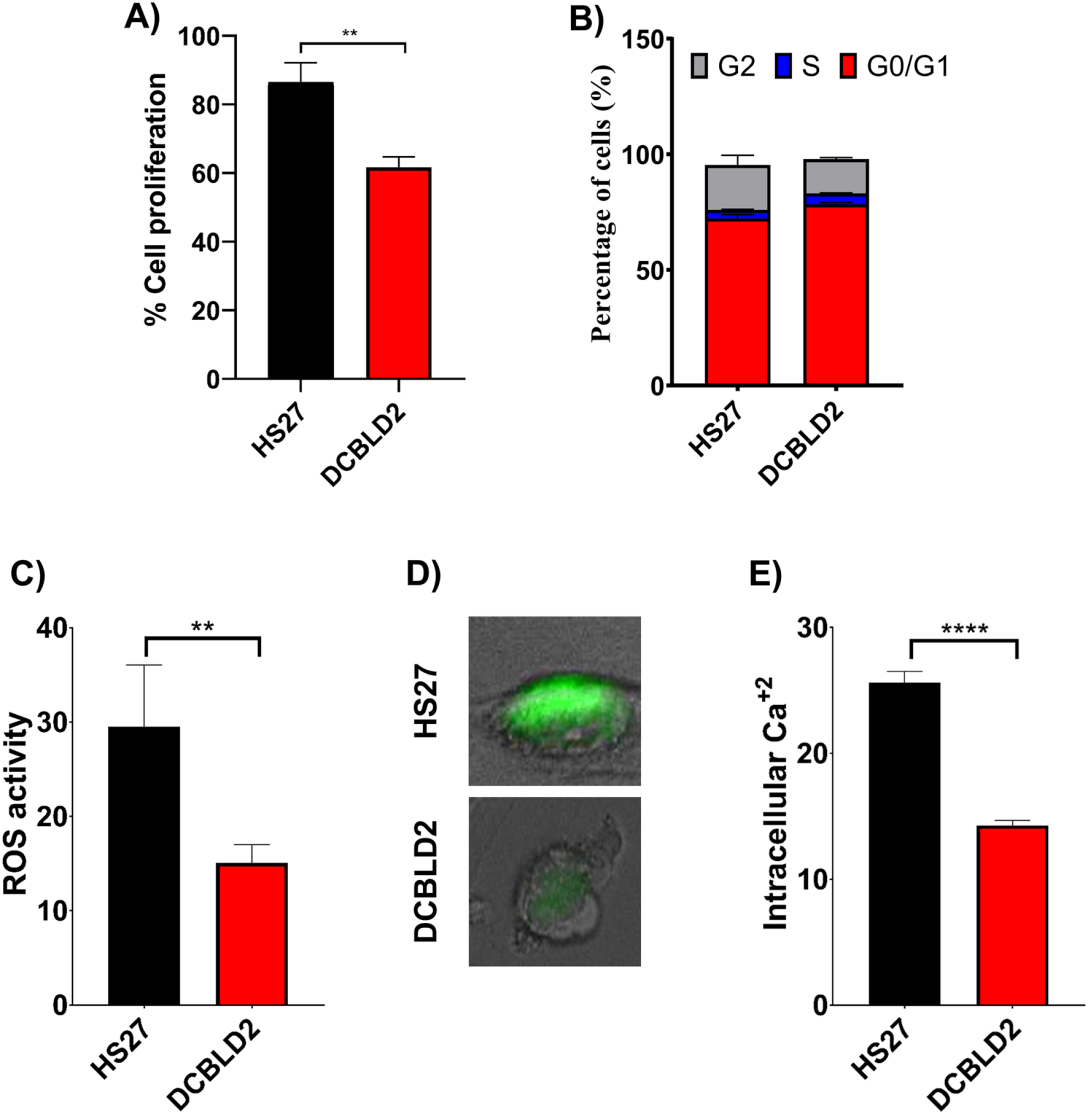
Functional characterization of the W27* variant in the *DCBLD2* gene. (**A**) The rate of cell proliferation in patient and control fibroblasts determined by the MTT assay. A significant reduction in cell proliferation was observed for the index’s fibroblasts with W27* (*P* = 0.0054). Error bars refer to the standard error of the mean of five technical repeats and four independent biological experiments (n = 4). (**B**) Quantification of the cell cycle distribution of W27* and normal control cells. Index fibroblasts with the DCBLD2 W27* variant had slight prolongation of G1 phase. Error bars refer to the standard error of the mean of two biological repeats. Student’s t-test using GraphPad Prism software. (**C**) Quantitation of reactive oxygen species (ROS) in the proband’s fibroblasts with the nonsense mutation and normal control fibroblasts performed using the cell-permeable fluorogenic probe 2′,7′-dichlorodihydrofluorescein diacetate (*p* = 0.0015). Data are shown as the mean value ± SD, and five independent biological repeats were performed (n = 5). (**D**) Visualization and localization of the intracellular calcium level in a single cell from the patient’s and control fibroblasts. (**E**) Quantification of the intracellular Ca2 + level in the index’s fibroblasts with W27* compared to the normal control (*p* < 0.0001). Error bars refer to the standard error of the mean of five technical repeats and four independent biological experiments (n = 5).

Given the notable feature of the *DCBLD*2 gene and its domain structure similarity with neuropilins, which are critical co-receptors in developmental processes, raises the likelihood that DCBLD2 might have a similar set of functions in a variety of biological functions, including regulation of cell growth and intracellular signalling cascades that regulate a variety of cellular activities^19^. Thus, cell proliferation and cell cycle assays were performed to evaluate the effect of the identified variant in the patient’s fibroblasts compared to normal cell. We examined the effect of the nonsense variant on cell proliferation and cell cycle progression in the proband’s skin fibroblasts compared to control cells. MTT assay quantification showed a significant reduction in the proliferation of proband cells compared with their normal counterparts (**P = 0.0054) (Fig. 3A). To determine the effect of the identified variant on the cell cycle phase distribution, flow cytometric analysis was performed. The quantification of the cell cycle profiles is shown in Fig. 3B. There was an increase in the percentage of cells in the G0/G1 cell cycle phase in the proband’s fibroblasts (78.4 ± 0.8) compared to the normal control (72.3 ± 1.6). Similarly, the percentage of patient fibroblasts in S phase (4.8 ± 0.1) was 3.8 ± 0.2% for the control fibroblasts. This reduction in the number of cells entering S phase and G2 phase is likely due to delayed cell cycle progression and is supported by the reduced proliferation of patient fibroblasts.

Overall, although the W27* variant may prevent the timely progression of cells through the G1/S boundary and S phase, there was no significant alteration in the cell cycle distribution in W27* cells compared to normal control cells.

### Examination of intracellular ROS and calcium levels in W27* cells

Oxidative stress is associated with cardiovascular tissue damage triggering cardiovascular pathologies, such as cardiomyopathy development^35^. These effects are mainly mediated by excessive ROS generation and a decrease in antioxidant responses in the heart and vasculature. Therefore, we detected the effect of the W27* variant on ROS levels in the proband’s skin fibroblasts compared with control cells. ROS were measured by performing a cell-based assay by using the cell-permeable fluorogenic probe DCF-DA. As expected, the results showed a disturbance in ROS generation with a significant reduction in patient fibroblasts in comparison to the control (*P* = 0.0015) (Fig. 3C). An increase in intracellular ROS generated by mitochondria or other enzymes mediates Ca2+ signalling dysfunction and consequently induces impairment and loss of functional myocytes and fibrosis development in the heart^36^. It was reported that abnormal oxidative stress induces intracellular Ca2+ dysregulation in cardiomyocytes^37^. Due to the involvement of Ca2+ in different clinical phenotypes of our patient and to determine the effect of the identified variant, we measured the intracellular Ca2+ level in patient skin fibroblasts and normal control cells. As shown in Fig. 3D, the basal level of intracellular calcium was significantly decreased in affected fibroblasts (*p* < 0.0001) compared to the control (Fig. 3E).

## Discussion

In this report, a novel variant in the *DCBLD2* gene associated with RCM, developmental delay, spasticity, and dysmorphic features was reported for the first time in a Saudi patient. WES analysis revealed the identification of two variants, namely, a heterozygous variant in *ACVRL1* (c.1285 G > T, p.V429L (GTG > TTG)) and a homozygous nonsense variant in *DCBLD2* (c.80 G > A, p.W27* (TGG > TAG)), to be associated with the patient’s phenotype. Although it is well known that pathogenic variants in *ACVRL1* are associated with autosomal dominant hereditary haemorrhagic telangiectasia type 2 (HHT2)^34^, the identified p.V429L variant was not described in the literature but is localized within a region with several HHT2-associated mutations. Missense mutations in the nearby residues (P424T/S/L/R, F425V/L, Y426C, D427V, and P433S/R) have been reported in the human gene mutation database in association with haemorrhagic telangiectasia^38^. *ACVRL1* gene mutations are also associated with a higher risk of pulmonary arterial hypertension^39^. All these findings support the functional importance of this region of the protein. Thus, the V429L variant was considered a strong candidate for a disease-causing mutation that may be associated with some of the patient’s reported intracranial haemorrhage, anaemia, and thrombocytopenia. However, the homozygous nonsense W27* variant in the *DCBLD2* gene has not been reported previously as a disease-causing mutation. Given the critical role of the *DCBLD2* gene in the regulation of cell growth, it is possible to consider it a candidate gene with a potential relationship with the phenotype. DCBLD2 encodes an endothelial and smooth muscle cell-derived transmembrane protein that plays a role in angiogenesis regulation. DCBLD2 knockout mice display reduced angiogenesis post-natally and have impaired blood flow recovery following arterial ligation^40^. *DCBLD2* is expressed in human platelet precursor cells, and studies in zebrafish indicate that *DCBLD2* may be involved in the inhibition of thrombus formation^20^. In this study, the molecular and functional characterization of the identified homozygous nonsense variant (p.W27*) in the *DCBLD2* gene that might be associated with lethal restrictive cardiomyopathy, developmental delay, spasticity and dysmorphic features was linked to multiple cellular processes for the first time.

The annotation and alignment of the identified variant (TGG > TAG), which creates a stop codon, revealed that W27* falls in an active regulatory site in the single sequences of the N-terminal region. Thus, this may have a major consequence on the *DCBLD2* transmembrane receptor function. It has been speculated that this stop codon in W27* may truncate the formation of all receptor domains, including the CUB domain, LCCL domain, and factor V/VIII homology domain in the extracellular N-terminus and the intracellular C-terminus, and thus might affect its cellular function. Subsequently, this will probably affect the simultaneous connection between receptors, kinases, substrates, and effectors. Each domain in the extracellular complement of *DCBLD2* has been shown to offer great flexibility to the receptor and allow it to play distinctive roles in cell proliferation and migration. The CUB domain has been involved in functions that include developmental patterning, tissue repair, and angiogenesis^41^. Although little is known about the LCCL domain, it has been proposed to be involved in lipopolysaccharide binding^42^. In contrast, the factor V/VIII domain is well characterized and critically involved in vascular coagulation^43^. Additionally, it will bind anionic phospholipids on platelets and endothelial cells^43^. Furthermore, the intracellular C-domain allows the recruitment of proteins that have phosphorylation-dependent binding domain motifs that recognize phosphoserine, phosphotyrosine, and phosphothreonine residues^44^. Seven possible binding motifs for the adaptor protein CT10 regulator of Kinas (Crk) and its homologue, CT10 regulator of Kinas-like (CrkL), have been identified in the intracellular domain of the *DCBLD2* scaffolding receptor^45^. *DCBLD2* has been shown to serve as a binding partner with Src homology domain 2 (SH2) of the CrkL signalling adaptor protein that subsequently allows the binding of different proteins and thus governs cellular changes^45^.

Due to sample limitations, the phenotype-genotype correlation to prove the pathogenicity of the identified variant has not been directly characterized. However, giving the structural domain similarity of DCBLD2 with neuropilins such as semaphorins, VEGF, and BAMBI, which are a specific VEGF, which are specific VEGF molecules that are critical co-receptors in developmental processes involved in neuronal migration, tumour angiogenesis, and progression^46^, raises the likelihood that DCBLD2 has a similar set of functions in cell growth and intracellular signalling cascades that regulate a variety of cellular activities. The role of DCBLD2 in regulation of cell growth is known and has been demonstrated previously in VSMC proliferation^18^. Herein, we have addressed the pathogenic effect of this nonsense variant in multiple cellular processes. Indeed, a significant reduction in the proliferation of the probands’ fibroblasts was observed compared to the normal control cells (**P = 0.0054) (Fig. 3B). Additionally, an increase in the percentage of cells in both the G0/G1 and S phases of the cell cycle in the patient fibroblasts compared to the normal control was displayed, indicating an arrest of the cell cycle progression (Fig. 3C). Several studies have revealed evidence regarding the importance of this protein in development and cellular processes^18,19,22^. Knocking down the ESDN level in VSMCs revealed a reduction in ESDN mRNA levels and consequently inhibited cell proliferation in HEK293 cells^19^. Similarly, it was demonstrated that confluence-induced growth inhibition and the subsequent cell cycle arrest in G0/G1 can also affect the expression and activity of a number of factors that play an important role in vascular remodelling^18^. Similarly, some semaphorins, such as semaphorin 3A (SEMA3A), semaphorin 3B (SEMA3B), and semaphorin 3F (SEMA3F), have been involved in the collapse of neuronal growth cones and in tumourigenesis^47,48^. Collectively, these results suggest a critical role for DCBLD2 in a wide variety of biological functions, including the intercellular receptor signalling pathway, regulation of cell growth, and wound healing.

Moreover, it is well known that the dysregulation of cellular ROS and Ca2+ homeostasis are usually associated with inflammation, endothelial dysfunction and remodelling, leading to vascular injuries and end organ damages. Interestingly, a significant reduction in the intracellular ROS and Ca2+ levels was found in the proband’s fibroblasts compared to a normal control. This indicates a dysregulation in ROS generation and Ca2+ distribution in the index’s fibroblasts, and may implicate the clinical phenotype observed in our patient. We cannot definitively extrapolate these results to the whole body, but it is shown that there is a calcium disturbance, and if this occurs within cardiomyocytes, it could be at least a major cause of the RCM characterized in the patient. It is well known that the overproduction of ROS results in oxidative stress (OS), which is considered a deleterious process. OS is involved in the damage of cell structures that causes the development of various pathological conditions, including kidney damage^49^, vascular injuries and hypertension^50^ and cardiomyopathy^51^. However, over the last 10 years, increasing evidence has revealed that ROS are not always considered harmful metabolic products; instead, various fundamental physiological functions of ROS in cell homeostasis have been revealed^52^. ROS, when strictly regulated, act as intracellular signalling molecules and are key players in the activation of different members of signalling cascades involved in the cell processes modulating proliferation and differentiation^53^. This occurs by modulating the activity of the oxidized targets, leading to the regulation of several normal physiological functions at the cellular level. At the cellular level, ROS play a critical role in modulating cell differentiation and proliferation. In addition, ROS contribute to complicated functions, such as blood pressure regulation^54^, cognitive functions^55^, cellular signalling mechanisms in the central nervous system^56^ and immune responses^57^. The dysregulation of ROS homeostasis found in the patient’s fibroblast indicating an imbalance at the physiologic level, which may disturb cellular metabolism, leading to cellular and molecular components abnormalities, which potentially explain, at least in part, the impairment in cell proliferation observed in our patient fibroblasts. Intracellular Ca2 + controls most of the vital intracellular processes, including cell division and proliferation and exocytosis, and modulates the activity of several enzymes. During pathophysiological condition, Ca2+ homeostasis is usually dysregulated^58^. Upregulation of intracellular Ca2+ in fibroblasts contributes to extracellular matrix (ECM) and collagen deposition, activation, and excessive proliferation^59^. Cardiomyocyte death and/or a decrease in contractility are the major factors involved in cardiomyopathy^60,61^, and the decrease in contractile force is a consequence of myofilament dysfunction or Ca2+ disturbance at the cellular level^62^. Taken together, these results suggest that Ca2+ and ROS dysregulation may be associated with the pathogenesis of developmental delay, RCM, and spasticity characterized in patients with the identified *DCBLD2* variant.

In summary, our analysis identifies a homozygous nonsense variant in *DCBLD2* that might be associated with RCM, developmental delay and dysmorphic features. However, this variant has been identified in a single family and may provide the first clue to the potential physiological function of the DCBLD2 protein in this important cardiological entity. In view of sample limitations, further research investigation will be crucial to understand the downstream signalling pathways in detail, which could help to explain the relationship between this variant and clinical phenotypes. However, this report adds to the ever-expanding landscape of genetic causes of RCM and increases our understanding of the cellular processes underlying this important cardiological entity.

## Abbreviations

CVD: Cardiovascular disease
HCM: Hypertrophic cardiomyopathy
DCM: Dilated cardiomyopathy
RCM: Restrictive cardiomyopathy
ESDN: Endothelial and smooth muscle cell-derived neuropilin-like protein
CLCP1: Charcot–Leyden crystal protein pseudogene 1
WES: Whole exome sequencing
